# Analysis of miR‐10a and *IFNG* Expression Before and After Treatment for Chronic *Schistosomiasis mansoni*

**DOI:** 10.1111/pim.70011

**Published:** 2025-06-05

**Authors:** Débora Nascimento da Nóbrega, Ana Virgínia Matos Sá Barreto, Roberta dos Santos Souza, Kleyton Palmeira do Ó, Raul Emídio de Lima, Ana Lúcia Coutinho Domingues, Edmundo Pessoa Lopes, Clarice Neuenschwander Lins de Morais, Elainne Christine de Souza Gomes, Luydson Richardson Silva Vasconcelos

**Affiliations:** ^1^ Instituto Aggeu Magalhães (IAM/Fiocruz) Recife Pernambuco Brazil; ^2^ Centro Universitário Maurício de Nassau (UNINASSAU) Recife Pernambuco Brazil; ^3^ Universidade Federal de Pernambuco, Hospital das Clínicas Recife Pernambuco Brazil

**Keywords:** gene expression regulation, interferon‐gamma, microRNA, praziquantel, schistosomiasis

## Abstract

Mir‐10a acts in signalling pathways regulating transcription, translation, and RNA‐mediated gene silencing, while IFNG acts in the T‐cell receptor signalling pathway. Thus, both can be considered potential targets for understanding regulatory processes in chronic inflammation in patients with schistosomiasis. Populations in endemic areas receive mass and indiscriminate praziquantel treatment, and yet patients often have a history of multiple infections. To investigate the regulatory and prognostic capacity of miR‐10 and IFNG in patient immunity, we evaluated the expression of miR‐10a and IFNG as biomarkers of inflammation and their correlation with praziquantel treatment. miR‐10a did not present evidence as a biomarker of inflammation in the therapeutic follow‐up in schistosomiasis. However, the levels of *IFNG* expression were significantly higher before treatment.

## 1 Introduction

In 1948 the World Health Organisation (WHO) recognised the relevance of schistosomiasis for Public Health. In 1980 Bayer and Merck Laboratories developed Praziquantel (PZQ), and the WHO subsequently recommended large‐scale treatment with PZQ in populations at risk of infection, regardless of whether they were infected or not, along with other complementary prevention measures [1, 2]. Although the use of PZQ has been recommended as a treatment for more than 40 years, in many endemic communities there is no success in eliminating the disease despite massive treatment efforts [3, 4].

At the beginning of infection, T helpers 1 cells induce a pro‐inflammatory response with the release of INF‐γ. Although levels of INF‐γ decrease with the advance of the disease as a result of increased anti‐inflammatory cytokines IL‐10 and TGF‐β released by Th2 [5]. Some studies have raised evidence that th1 response and IFN‐γ levels may increase after treatment in acute patients, when stimulated with antibodies, and contribute to resistance to new infections [6, 7, 8, 9]. Unlike these studies, in this paper, we included chronic patients with liver fibrosis and a history of previous infections, analysing before and after treatment.

Posttranscriptional regulators such as microRNAs (miRNA) are responsible for individual variation in the susceptibility of infections and chronicity of parasitic diseases [10]. Hsa‐miR‐10a is an IFN‐γ antagonist and inhibits the Th1 response produced by FOXP3+ regulatory T cells [11, 12], which were also associated with increased TGFβ‐1 and activation of the TGFβ pathway, which corroborates hepatic fibrosis [13, 14].

For this reason, the aim of this study was to evaluate the expression of miR‐10a and *IFNG* before and after treatment with praziquantel *for Schistosoma mansoni* in chronic infection. The hypothesis was that miR‐10 could be expressed, reducing IFN‐γ expression and thus slowing a pro‐inflammatory response in patients due to their chronic infectious status.

However, miR‐10 expression in Peripheral Blood Mononuclear Cells (PBMCs) was very low both before and after treatment, suggesting that miR‐10 is not significant in regulating the inflammatory response in schistosomiasis via PBMCs. On the other hand, this study shows that even in a chronic state, IFN‐γ expression remains significant and there is no compensation after treatment as an immune resistance mechanism.

## 2 Methods

### 2.1 Study Population

The samples were obtained from the population survey conducted in Porto de Galinhas (*n* = 45), in the state of Pernambuco (Brazil), by the Reference Service in Schistosomiasis from Instituto Aggeu Magalhães (IAM—Fiocruz). The 45 samples were stored in the biorepository; 30 belonged to the group of patients with a diagnosis for *S. mansoni* through Kato‐Katz, and 15 belonged to the group of patients without infection (controls).

Patients who presented stool samples positive for *S. mansoni* received praziquantel treatment and samples were collected before and after 90 days of treatment. Liver evaluation was obtained by abdominal ultrasound scan carried out by the same examiner with experience with this disease, and the Niamey classification was used for liver fibrosis measurement [15].

### 2.2 Biochemical Markers Dosage of Liver Damage

Serum samples were measured with the support of Hospital das Clínicas' Laboratory (Pernambuco—Brazil) for the following biochemical markers of liver damage: Direct Bilirubin (DB) and Total Bilirubin (TB), Alkaline Phosphatase (ALP), Aspartate Aminotransferase (AST), Alanine Aminotransferase (ALT), and Gamma Glutamyl Transferase (GGT).

### 2.3 RNA Extraction, cDNA Synthesis and Expression Assay

PBMCs were isolated using the Ficoll Paque gradient method. Total RNA extraction in these isolated cells was performed with Trizol (Invitrogen). After purification of total RNA, cDNA was prepared with 10 ng of total RNA (in 5 μl) using the TaqMan microRNA Reverse Transcription Kit with a primer specific to miR‐10a. For cDNA synthesis of *IFNG*, 2000 ng (2 μg) of RNA input was used in a 20 μl reaction, using primers randomised using the High‐Capacity cDNA Reverse Transcription Kit, following the manufacturer's protocol. The differential expression of miR‐10a and *IFNG* was evaluated in patients with schistosomiasis and controls, using Taqman microRNA Assay, following the manufacturer's instructions in the QuantStudio 5 Real‐Time PCR System (Applied Biosystem). Both primers and probes were obtained for Taqman Gene Expression Assays (ID MIR10A: Hs04231458_s1 and ID IFNG: Hs00989291_m1). The data were normalised using β‐actin as an endogenous control. The relative expression was calculated by the 2^−ΔΔCt^ method.

### 2.4 Statistical Analyses

For the relative expression data of miR‐10a, the calculation of 2^−ΔΔCt^ was used for relative quantification of the expression that can have modified values depending on the group chosen as the reference for analysis. First, the group ‘Before Treatment’ was used as a reference; subsequently, the control group (patients negative for *S. mansoni* ) served as a reference for the other groups.

Shapiro–Wilk test was used for variable distribution analysis. In the paired samples to compare the groups ‘Before Treatment’ and ‘After Treatment’, the Wilcoxon matched pairs signed rank test (two‐tailed) was used. For the control group, unpaired *T* test was used. These analyses were performed using GraphPad Prism Software version 5. For bivariate correlation analysis, the IBM SPSS Statistics v. 28.0.1.1 and the Spearman Correlation Coefficient (two‐tailed) were used.

### 2.5 Ethical Considerations

This project was approved by the Research Ethics Committee of the Aggeu Magalhães Institute (CEP/IAM) CAAE: 38302320.8.0000.5190.

## 3 Results

Table 1 describes the demographic characteristics of the patients, the previous diagnosis of schistosomiasis, the history of previous treatment against schistosomiasis, parasitic load, as well as the ultrasound findings of the 45 individuals.

**TABLE 1:** Clinical and epidemiological characteristics of the patients in the study.

Clinical characteristics	Patients positive for *S. mansoni N* = 30	Patients negative for *S. mansoni N* = 15
Age [Years (minimum–maximum)]	32.5 (9–69)	32 (12–58)
Gender		
Male [*n*(%)]	20 (66.7%)	8 (53.4%)
Female [*n*(%)]	10 (33.3%)	7 (46.7%)
Previous infection [*n* (%)]	7 (23.3%)	5 (33.4%)
Previous treatment [*n* (%)]	8 (26.7%)	11 (73.4%)
Parasitic load [epg (minimum–maximum)]	36 (1–1572)	—
Fibrosis pattern (niamey)		
A‐B [*n* (%)]	5 (16.7%)	9 (60%)
C‐D/DC [*n* (%)]	23 (76.7%)	6 (40%)
E [*n* (%)]	2 (6.6%)	0
Portal vein diameter (cm)	0.90 (95% CI: 0.85–0.96)	0.88 (95% CI: 0.81–0.94)

*Note:* A‐B = Without/minimal fibrosis; C = Peripheral fibrosis; D = Central fibrosis; DC = Central and Peripheral fibrosis; E = Hyperechoic patches expanding from the portal vessels into the parenchyma.

Because it is an endemic area for schistosomiasis, 23.3% of the patients with *S. mansoni* (*n* = 30) reported having presented previous infection in the years prior to the survey conducted by the IAM‐Fiocruz, and more than 30% of the control patients with negative results in the parasitological, molecular, and immunological tests (*n* = 15). These patients also received treatment in previous years through the SANAR program of the Pernambuco State Secretariat. Interestingly, a large proportion of patients negative for *S. mansoni* (73.4%) had received previous treatment, having presented previous infection or not (Table 1).

**FIGURE 1:**
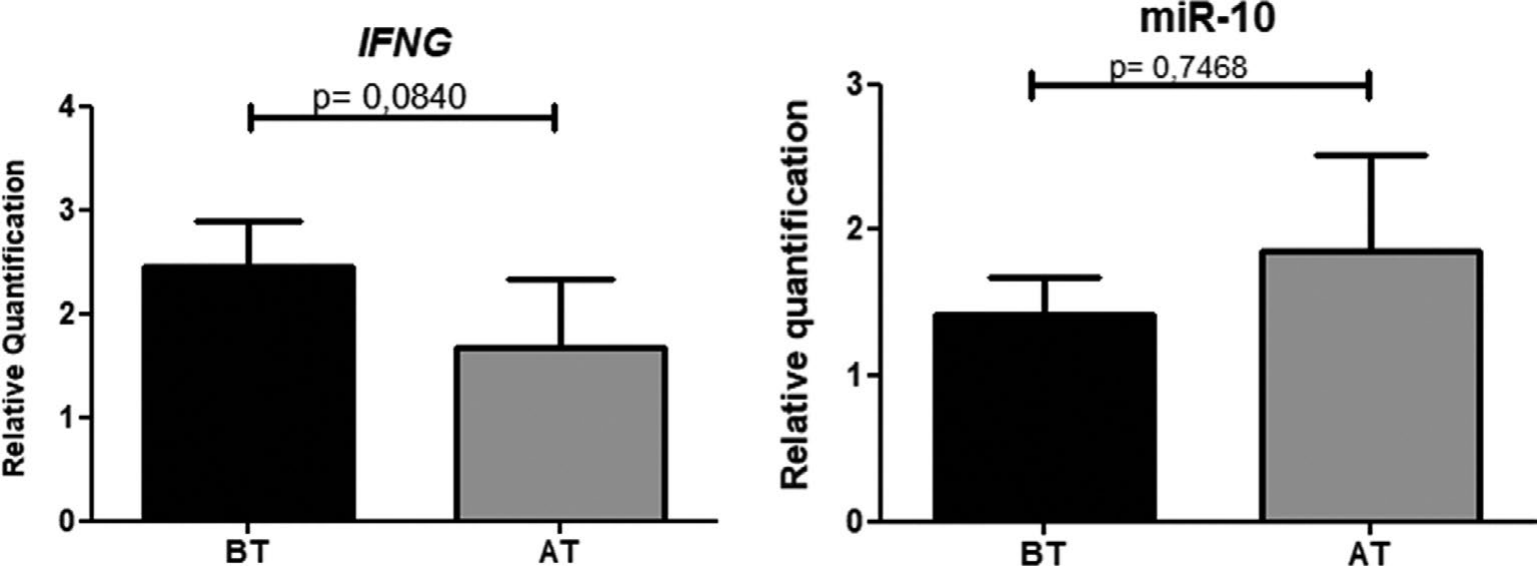
Relative quantification (2^−ΔΔct^) of the expression of *IFNG* and miR‐10a. Wilcoxon Test. AT, after treatment; BT, before treatment.

When using the group addressed as ‘Before Treatment’ as a reference, the relative expression of *IFNG* before Praziquantel treatment was higher than after 90 days, but there was no significant difference (*p* = 0.2741). The relative expression of miR‐10a after treatment was higher; however, this difference was also not statistically significant (Figure 1).

**FIGURE 2:**
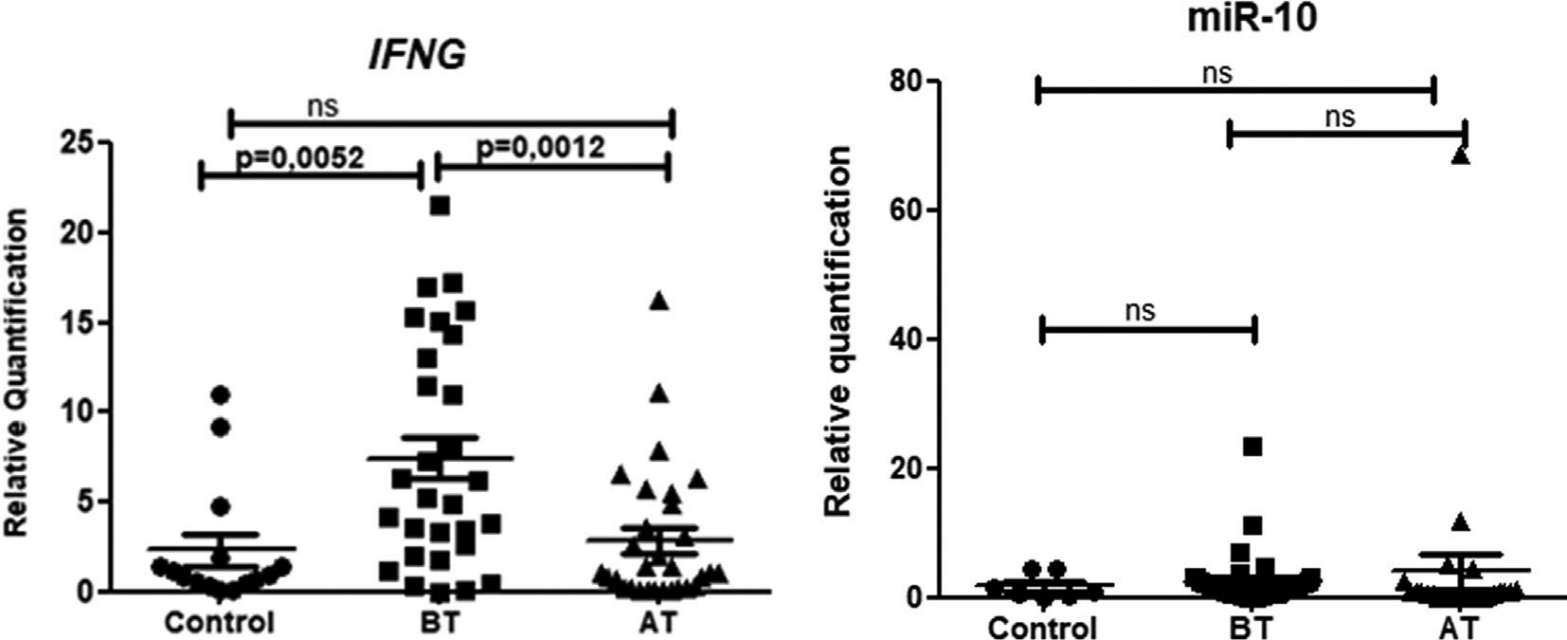
Relative quantification (2^−ΔΔct^) of the expression of *IFNG* and miR‐10a before and after treatment with reference to the expression of control samples. Unpaired *t* test. AT, after treatment; BT, before treatment; ns, non‐significant.

On the other hand, the calculation 2^−ΔΔCt^ was performed assuming the relative expression of non‐infected patients (*n* = 15) as a reference, *IFNG* values before Praziquantel therapy were significantly higher than after treatment, see Figure 2 (*p* = 0.005).

There was no correlation between miR‐10a and parasitic load, treatment, and hepatic fibrosis, but it was significantly correlated with *IFNG*, presenting a positive correlation (Table 2). However, the Spearman coefficient value indicates that this correlation is weak (*R* = 0.482). There was also a negative but weak correlation between IFNG and treatment (*p* = 0.043; *R* = −0.228).

**TABLE 2:** Correlation test between IFNG and miR‐10a in patients with schistosomiasis before treatment (*n* = 30) with parasitic load, liver fibrosis, previous treatment, and *IFNG*.

Variables	*IFNG*		miR‐10a	
	*R*	*p*	*R*	*p*
Parasitic load	0.079	0.622	0.235	0.138
Liver fibrosis	0.020	0.904	−0.151	0.354
Treatment	−0.228	**0.043**	−0.210	0.091
*IFNG*	1	—	0.482	**0.001**

*Note:* Spearman test. The significance results (*p* < 0.05) are presented in bold.

There was no correlation between miR‐10a and IFNG with serum biochemical markers of liver inflammation (Table 3).

**TABLE 3:** Correlation between biochemical parameters and *INFG*/miR‐10a in patients with schistosomiasis before treatment (*n* = 30).

Biochemical parameters	*IFNG*		miR‐10a	
	*R*	*p*	*R*	*p*
ALP (U/L)	−0.0819	0.6972	0.1291	0.5384
GGT (mg/dL)	−0.0894	0.6707	−0.1042	0.6203
AST (U/L)	−0.2333	0.2616	0.1310	0.5326
ALT (U/L)	−0.2180	0.2952	−0.1806	0.3877
BT (mg/dL)	−0.1602	0.4444	0.3166	0.1232
BD (mg/dL)	−0.0970	0.6446	0.1163	0.5800

*Note:* Pearson test (two‐tailed).

## 4 Discussion

In this study, it was seen that IFN‐Υ levels are significantly higher in chronic patients prior to treatment with praziquantel than 90 days after treatment or than controls (Figure 2). However, there was no correlation between IFN‐Υ and liver fibrosis pattern (Table 2) or biochemical parameters (Table 3); this can be attributed to the small number of samples when the comparison groups were distributed.

Studies show that IFN‐Υ is capable of preventing the progression of fibrosis by stimulating mesenchymal stem cells (MSCs) that prevent fibroblast activation and extracellular matrix expansion [16]. IFN‐Υ is also capable of inhibiting TGF‐β signalling, through phosphorylation and activation of the transcription factor Stat1, which in turn induces the expression of SMAD7 responsible for inhibiting the TGF‐β pathway [16, 17].

In our results we did not see a correlation between miR‐10a and fibrosis, but in the literature, associations of this microRNA in the development of fibrosis were found. An experimental study using hepatic fibroblasts from 20 mice with carbon tetrachloride‐induced fibrosis showed that miR‐10a is highly expressed in this group when compared to the expression of 20 liver tissue samples from controls (−7.84 ± 1.38 vs. −9.97 ± 1.59, *p* < 0.05) [13]. In addition, the authors revealed that miR‐10a is capable of increasing fibroblast proliferation. When fibroblasts were transferred to culture with the addition of mimicked miR‐10a, the concentration of TGFβ1 increased significantly, while the expression of SMAD7 (TGF‐β inhibitor) decreased [13].

Similarly, in another study with intestinal mucosa culture of patients with Crohn's disease (inflammatory bowel disease), TGF‐β was able to increase the expression of miR‐10a while IFN‐Υ and TNF had antagonistic effects, inhibiting the expression of miR‐10a [12].

Cabantous et al. performed RNAseq with 22 samples of liver biopsy from patients infected with *Schistosoma japonicum* (in China), presenting mild or absent peripheral fibrosis, and central fibrosis in the left lobe of mild to moderate thickness, and found significantly *upregulated* miR‐10a in patients with fibrosis compared to control liver samples (donors in France) [14].

Unlike the study conducted by Cabantous et al. our study is the first to investigate the expression of miR‐10a in PBMCs of patients with *Schistosomiasis mansoni* and compare with the times before and after Praziquantel administration [14]. A previous study investigated the expression profile in T cells isolated from PBMCs and liver tissue of mice infected with *S. japonicum* . According to the authors, miRNA‐10a is co‐detected in T cells originating from PBMCs and the liver, but has different expressions, being more expressed in the liver [18].

Thus, investigating the correlation of miR‐10a with fibrosis in liver cells may yield better results than in PBMCs. However, the number of patients in this study is small to draw conclusions about the role of miR‐10a in fibrosis and immune response regulation during *S. mansoni* infection. Other circulating microRNAs in serum and exosomes have also been described in the literature as biomarkers of liver fibrosis [19, 20].

A study by Kelada et al. [11] showed that during acute inflammation caused by *Leishmania major*, the downregulation of miR‐10a promotes recruitment of Foxp3+ Treg cells, which positively regulate a set of miR‐10a target genes. Among these targets, *Nr4a3* stands out for inducing Foxp3 expression. In the same study, it was observed that treatment of natural and induced Treg cells with IL‐12/IFN‐γ reduced the expression of Creg and miR‐10a, while inducing a fourfold increase in IFN‐γ release. When combined with overexpression of miR‐10a mimics, these cells increased IFN‐γ production by 40 times, demonstrating that miR‐10a is regulated by type 1 immune responses.

Interestingly, our results showed positive regulation of IFNG expression during *S. mansoni* infection compared to controls, while miR‐10a levels did not differ. Therefore, we can speculate that IFNG expression may have suppressed miR‐10a expression and also may indicate an acute inflammatory status, even though these patients had chronic liver damage.

## 5 Conclusion

In this study, miR‐10a did not present differences in PBMCs of patients with chronic schistosomiasis after Praziquantel therapy, however *IFNG* expression levels decreased significantly.

## Author Contributions

**Débora Nascimento da Nóbrega:** performed the experiments and analyses, prepared and edited the manuscript. **Ana Virgínia Matos Sá Barreto:** performed the experiments and analyses. **Roberta dos Santos Souza:** performed the experiments; edited the manuscript. **Kleyton Palmeira do Ó:** performed the experiments. **Raul Emídio de Lima:** performed the experiments. **Ana Lúcia Coutinho Domingues:** edited the manuscript. **Edmundo Pessoa Lopes:** edited the manuscript. **Clarice Neuenschwander Lins de Morais:** edited the manuscript. **Elainne Christine de Souza Gomes:** edited the manuscript. **Luydson Richardson Silva Vasconcelos:** edited the manuscript.

## Conflicts of Interest

The authors declare no conflicts of interest.

## Peer Review

The peer review history for this article is available at https://www.webofscience.com/api/gateway/wos/peer‐review/10.1111/pim.70011.

## Data Availability

The data that support the findings of this study are available on request from the corresponding author. The data are not publicly available due to privacy or ethical restrictions.
